# Classification of Watermelon Seeds Using Morphological Patterns of X-ray Imaging: A Comparison of Conventional Machine Learning and Deep Learning

**DOI:** 10.3390/s20236753

**Published:** 2020-11-26

**Authors:** Mohammed Raju Ahmed, Jannat Yasmin, Eunsung Park, Geonwoo Kim, Moon S. Kim, Collins Wakholi, Changyeun Mo, Byoung-Kwan Cho

**Affiliations:** 1 Department of Biosystems Machinery Engineering, College of Agricultural and Life Science, Chungnam National University, 99 Daehak-ro, Yuseong-gu, Daejeon 34134, Korea; mrahmed@o.cnu.ac.kr (M.R.A.); yasminjannat@o.cnu.ac.kr (J.Y.); moon.kim@usda.gov (E.P.); wcoln@o.cnu.ac.kr (C.W.); 2 Environmental Microbial and Food Safety Laboratory, Agricultural Research Service, United States Department of Agriculture, Powder Mill Road, BARC-East, Bldg 303, Beltsville, MD 20705, USA; Moon.Kim@ars.usda.gov; 3 Department of Biosystems Engineering, College of Agriculture and Life Sciences, Kangwon National University, Chuncheon 24341, Korea; cymoh100@kangwon.ac.kr; 4 Department of Smart Agriculture System, College of Agricultural and Life Science, Chungnam National University, Daejeon 34134, Korea

**Keywords:** seed quality, watermelon, X-ray imaging, image analysis, transfer learning, nondestructive measurement

## Abstract

In this study, conventional machine learning and deep leaning approaches were evaluated using X-ray imaging techniques for investigating the internal parameters (endosperm and air space) of three cultivars of watermelon seed. In the conventional machine learning, six types of image features were extracted after applying different types of image preprocessing, such as image intensity and contrast enhancement, and noise reduction. The sequential forward selection (SFS) method and Fisher objective function were used as the search strategy and feature optimization. Three classifiers were tested (linear discriminant analysis (LDA), quadratic discriminant analysis (QDA), and k-nearest neighbors algorithm (KNN)) to find the best performer. On the other hand, in the transfer learning (deep learning) approaches, simple ConvNet, AlexNet, VGG-19, ResNet-50, and ResNet-101 were used to train the dataset and class prediction of the seed. For the supervised model development (both conventional machine learning and deep learning), the germination test results of the samples were used where the seeds were divided into two classes: (1) normal viable seeds and (2) nonviable and abnormal viable seeds. In the conventional classification, 83.6% accuracy was obtained by LDA using 48 features. ResNet-50 performed better than other transfer learning architectures, with an 87.3% accuracy which was the highest accuracy in all classification models. The findings of this study manifested that transfer learning is a constructive strategy for classifying seeds by analyzing their morphology, where X-ray imaging can be adopted as a potential imaging technique.

## 1. Introduction

Watermelon (*Citrullus lanatus* (Thunb.)) is a popular delicious and refreshing fruit that is consumed all over the world and is a great source of vitamins and minerals. The cultivation of watermelon starts with the sowing of seeds in the soil and the production of seedlings. Therefore, for producing healthy and high-quality seedlings, seed quality inspection is a concern for both watermelon growers and seed companies.

**Figure 1:**
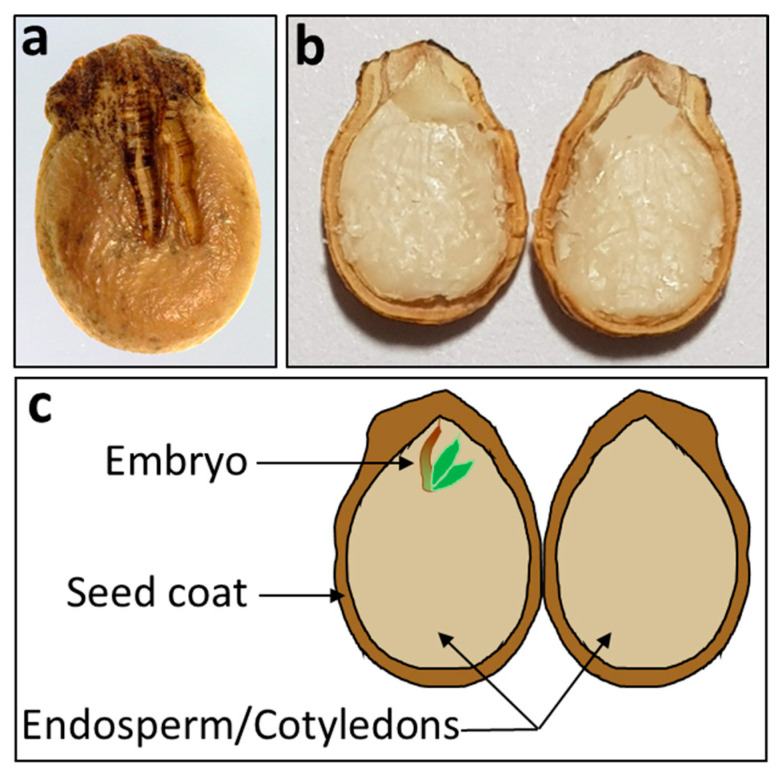
Basic components of a dicot seed (watermelon). (**a**) external view and (**b**) internal view of seed; (**c**) illustration of major parts of seed.

A watermelon seed is classified as a dicotyledon seed containing three basic components: (1) a seed coat, (2) an embryo, and (3) endosperm (Figure 1). Each component plays a vital role for ensuring successful germination. The seed coat protects the inner parts of the seed from the outside environment, the embryo is a tiny plant that grows upwards, and endosperm stores food to provide the necessary nutrients during germination [1]. To inspect the inner components of a seed, X-ray imaging can be used as a feasible resource because it is fast, reliable, and nondestructive compared to ordinary seed quality testing methods, such as the tetrazolium test (TZ test), pepper germination test, and embryo excision test [2,3].

X-ray imaging was programmed as an official method for inspecting the internal insect infestation in grains or seeds by the Association of Analytical Chemists (AOAC) in 1961 (AOAC, 1980) [4]. Since then, X-rays have been used for the internal quality assessment of various types of agricultural products, such as fruits [5], grains [6,7] and food [8]. Several studies have been conducted using X-ray-based quality monitoring systems to examine the internal component structures of seeds, which effect the germination power of the seed. To identify filled, partially filled, and embryonic tissues in guayule seeds, X-ray imaging analysis was used by Jorge and Ray [9]. The chi-square result showed that filled seeds had a higher germination rate than partially filled and unfilled seeds. The internal morphology of *Platypodium elegans* Vog. seeds was evaluated through X-rays and the result was verified with a germination test by Paradelo Gomes et al. [10]. In addition, X-ray imaging has also been used to inspect the internal crack detection of bean seeds [11], for seed viability evaluations based on filled or unfilled seeds of wild species from Saudi Arabia [12], and to assess the morphology of tomato seeds [13]. However, a classification model development is required to use the X-ray imaging technique combined with machine vision and pattern recognition, which would be beneficial for practical use.

In recent years, typical machine vision classification techniques have been replaced with deep learning [14] because of benefits such as the lack of redundancy of handcrafted image feature extraction and better classification results [15,16,17]. Training the neural network architectures which are formed of different nonlinear processing layers is termed deep learning. For machine vision tasks, particularly for image processing, the convolutional neural network (CNN or ConvNet) has become the standard neural network [14]. To solve many agricultural problems, deep learning approaches have been used by several researchers. From leaf vain morphological patterns, plant identification and classification was studied by Grinblat et al. [18] by applying ConvNets. Sun et al. [19] used a residual network (ResNet), which is the most common ConvNet architecture for plant species classification, and obtained a 91.8% accuracy. Six network architectures, namely AlexNet, VGG-19, GoogLeNet, ResNet-50, ResNet-101, and Inception-v3, were tested by Suh et al. [20] for sugar beet and volunteer potato classification. A self-design ConvNet and a residual network (ResNet) were used to identify cotton seed varieties, where the input images were acquired from hyperspectral imaging by Zhu et al. [21]. Previously, a similar procedure was applied for rice variety identification and obtained an accuracy of around 90% [22]. These studies indicate that deep learning can be adopted as an adequate classification technique to classify seeds based on their morphological patterns exhibited in X-ray images.

The objective of this study is to classify naturally aged watermelon seeds based on their morphology related to germination. The novelty of this study is the use of X-ray imaging and deep learning in combination to classify seeds and the structure of their internal components, which directly affect the germination ability. In the deep learning approaches, the transfer learning of the mostly common deep learning networks such as simple ConvNet, AlexNet, VGG-19, ResNet-50, and ResNet-101 architectures were adopted for classification to find the best performance. Developed conventional linear discriminant analysis (LDA), quadratic discriminant analysis (QDA), and k-nearest neighbors algorithm (KNN) models from handcrafted image feature extraction, such as local binary pattern (LBP), Gabor, local Fast Fourier Transform (FFT), texture, contrast, and Haralick textural (Tx) features, were used for comparisons with the deep learning model results. Finally, custom-built software was developed for X-ray image analysis, model development, and predicting classes based on new samples, which could be useful for non-destructive seed quality inspection.

## 2. Materials and Methods

### 2.1. Sample Collection and Preparation

**Figure 2:**
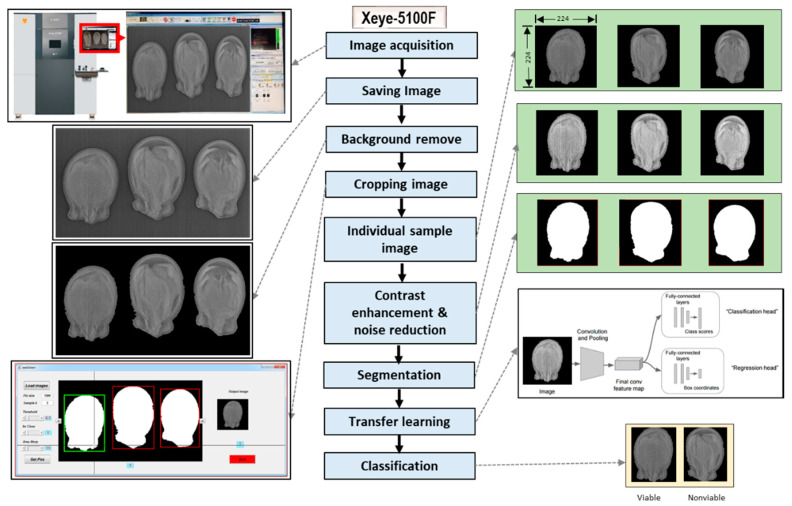
Illustration of the working procedure to develop the classification model for watermelon seed quality based on their morphological patterns.

Figure 2 shows the conceptual diagram for classification model development using transfer learning from the acquired X-ray images. For the experiment, three watermelon seed samples (Leehyunglim, Sambaechea, and Choiganggul) were obtained from the Korea Seed and Variety Service (KSVS) institute located in Kimcheon, Republic of Korea. The samples were stored for 6 years in sealed plastic containers at about 5 °C and 40% relative humidity (RH). After acquiring the seed samples from the institute, they were stored in a refrigerator at 4 °C until experiments. A total of 1800 seeds (600 seeds from each cultivars) were randomly chosen from the batch for taking X-ray images of the seeds.

### 2.2. Image Acquisition

An X-ray imaging system (Xeye-5100F, SEC, Suwon, Korea) with control software was used to take the X-ray 2D projection images of the samples. The X-ray source for the inspection system was a 10–100 kV micro-focus closed tube (5 µm focal spot size) and high-resolution detector. In addition, a high magnification power and four-axis manipulator configuration (X, Y, Z, and tilt) were integrated with the X-ray inspection system. For the image acquisition, a custom-made plastic transparent seed plate was used for 90 seed samples. To acquire magnified seed images of each cultivars, three samples were taken for every scan. The time for taking images was distinctly nominal because the exposure time of the X-ray was 0.05 s. The obtained images were preliminarily stored in bmp image format with a default size (2304 × 1300 pixels, width × height). The X-ray imaging parameters were summarized in Table 1. After the sample image acquisition, the seeds were placed in 96-well plates and stored in a refrigerator until the further germination test. A soft X-ray was used for generating images with a short radiation exposure time [23]. The used samples received less X-ray emission energy because of the magnification process during image acquisition to prevent the deterioration of seed quality.

### 2.3. Germination Test

To ensure the viability of the seed samples, the standard germination tests of international seed testing association (ISTA) were performed. The seeds were put in germination paper and folded, and they were stored in a germination chamber at 25 °C and 90% relative humidity for 14 days. The germination test information was used to develop supervised classification model.

### 2.4. Image Preprocessing

In this study, for the image processing, image cropping, resizing, masking, feature extraction, statistical computation, and classification were performed by Matlab software (The MathWorks, Natrick, MA, USA). To accomplish this, a personal computer system equipped with a 3.20 GHz Intel Core i5-4570 processor (CPU), 16 GB random access memory (RAM, type: DDR3), 1 GB NVIDIA GeForce GTX 650 graphics processing unit (GPU), and 64-bit Windows 10 Enterprise operating system (OS) was used and the results for conventional classification was evaluated. The specification of the transfer learning architecture-operated computer system was composed of CPU- Intel Core i9-7900X processor (10 core 20 threads), RAM-8 × 16 GB DDR4 (total 128 GB), GPU-4 × 8 GB NVIDIA RTX 2070 (total 32 GB), and OS-Ubuntu 18.04 LTS.

### 2.5. Image Cropping

In the raw format of the image, three samples were captured in each scan. Therefore, image cropping was used to produce individual sample images. A MATLAB-based custom-built algorithm was used for the cropping operation (Figure 2). Each sample image was saved in png format with a dimension of 224 × 224 pixels. Later, the cropped images were used for typical feature extraction and transfer learning.

### 2.6. Image Masking and Quality Enhancement

Image masking was applied on the selected region of interest (ROI) from the images where the whole seed was nominated as ROI, and the background was removed after ROI selection. In the masking process, ROI was selected as the foreground in which pixel values were considered as 1, and the background pixels were counted as 0. For masking, the balanced histogram thresholding method [24] was used, where Otsu’s method [25] was applied to determine the optimum threshold value. For quality enhancement of the images, maximum (max) normalization and standard deviation scaling was applied for intensity adjustment, and median filtering was used for noise reduction. At first, max normalization was applied on the cropped image to produce a set of images called set_A. Then standard deviation scaling was applied on set_A to generate set_B. Lastly, a 3 × 3 median filter was applied on set_B to produce set_C. Thus, a total of 3 sets of images were generated for image feature extraction.

### 2.7. Feature Extraction, Selection, and Classification

Six types of image features (basic intensity (6), local binary patterns (LBP) (234), Gabor intensity (67), basic contrast (5), Haralick texture (28), and local Fourier transform (FFT) texture features (8)) were extracted from the X-ray images for conventional machine learning classification. Total features number obtained from each image was 348. Three sets of images were used for a single sample; therefore, a total of 1044 features were extracted by individual seed images. The sequential forward selection (SFS) method was applied for search strategy while the Fisher score (J(W)) objective functions were used for the best feature selection. The most common classifiers such as linear discriminant analysis (LDA), quadratic discriminant analysis (QDA), and (4) the k-nearest neighbor algorithm (KNN), were tested with a 10-fold stratified cross-validation to find the best performer. The germination test results were adopted to construct supervised groups for the tested classifiers. Details of the image processing segments are available in Ahmed et al. [26].

### 2.8. Transfer Learning

The objective of transfer learning is to overcome the shortage of trained data and save time by transferring image features extracted from the pre-trained Nets [27,28]. In real world applications, transfer learning has shown promising success [18,29,30]. ConvNet architectures are one of the well-known deep learning structures which are specially designed for extracting image features, classification, and regression [18,21]. ConvNets have achieved good performances in various tasks, especially in computer vision. Typically, a ConvNet consists of three types of layers: convolution layers for image feature extraction, pooling layers for extracted feature compression, and fully-connected layers for classification.

**Figure 3:**
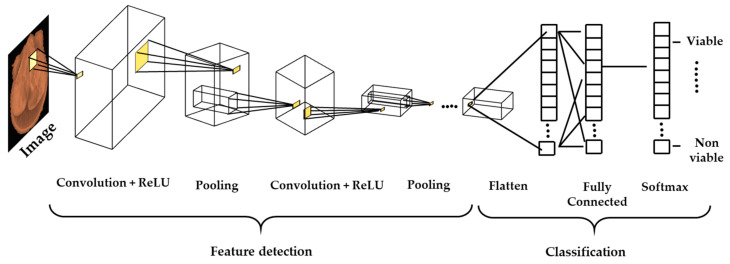
A ConvNets sequence to classify seeds into 2 classes.

In this study, five types of modified ConvNet architectures (simple ConvNet, AlexNet, VGG-19, ResNet-50, and ResNet-101) were used for the binary classification of X-ray seed images to find the best performer. In the ConvNet architectures, first, feature maps were produced by the convolution operations between the input images and a filter bank of square-sized maps. Each filter had a bounded size associated with a small receptive field in the input image. A rectified linear unit (ReLU) function was applied to all feature maps for the second transformation. The third transformation is called the pooling stage, where each map was divided into a set of non-overlapping square neighborhoods. From each neighborhood, this transformation only retains the maximum value. A softmax function that acted as a classifier was the last layer in this network. This layer was fully connected to all output feature maps of the last convolutional layer. It returned the estimated probability of each class, given a concrete sample. Figure 3 shows the ConvNet sequence employed to classify objects.

To create the image dataset, a total of 1282 images (641 normal viable and 641 nonviable and abnormal viable) were used. From each group, a similar sample number was used to create the dataset for eliminating the bias of a particular group in the classification result. Deep learning neural networks require big datasets in order to improve the performance and ability of the model to generalize. Therefore, each image from the dataset was augmented in six variations (rotation, reflection, translation (x, y), and scaling (x, y)), to create the final dataset that included 7692 images. The entire dataset was divided in two parts, where the training and validation parts contained 70% and 30% randomly selected samples from the dataset, respectively. Later, the validation set was split 50:50 to produce validation and test datasets, where the test dataset was used to observe the model behavior with an unknown sample. Hence, the dataset was divided into training/validation/test at a ratio of 70:15:15. In the simple shallow ConvNet architectures, images were input as grayscale images (one channel); however, other architectures are specially designed to handle color images (three channels: red, green, and blue). Therefore, the input images were converted into pseudo colored images and then used in the other architectures. Simple ConvNet architectures were developed with ReLU for non-linear activation and max-pooling for down-sampling functions to improve the training time efficiency, followed by a fully-connected layer (FC layer) and softmax layer, which were originally designed for the classification of 10 classes. Therefore, the last two layers were redesigned for classifying binary class. The used filter size was 3 × 3 with stride value 1.

AlexNet is one of the early deep network architectures where the initial layers are convolutional layers followed by ReLU and max-pooling. The last three layers are consisted of two fully-connected (FC) layers each with a 4096-dimensional activation vector and softmax layer with 1000 activation neurons for producing 1000 classes classification. To produce binary class output, last two layers (FC land softmax) were modified to fine-tuned with training images of viable and nonviable watermelon seeds. In VGG-19 architectures, the number of the parameters of the network were reduced by using the 3 × 3 filter in the convolution layers and max-pooling was used between convolution layers to reduce the network volume [31]. The last three layers consisted of two FC layers with 4096-dimensional activation vector and a softmax layer. For two class inspection, last two layers (FC and Softmax) were redesigned in the VGG-19 network. ResNet contains several residual blocks to provide a shortcut connection between layers which enhance the performance to train hundreds of layers in the network. The primary objective of ResNet design is large-scale data analysis using many different numbers of layers, for example ResNet-50 and ResNet-101contain 50 and 101 convolutional layers, respectively, where the end layer includes a FC layer. In the transfer learning of ResNet-50 and ResNet-101 of this study, the last three layers—convolution layers, fully-connected (FC) layers, and softmax—of the architectures were removed as they were designed to classify 1000 classes (originally trained with the ImageNet dataset), and constructed new layers (FC and softmax) were added to classify the two classes of seed.

### 2.9. Analysis Software

**Figure 4:**
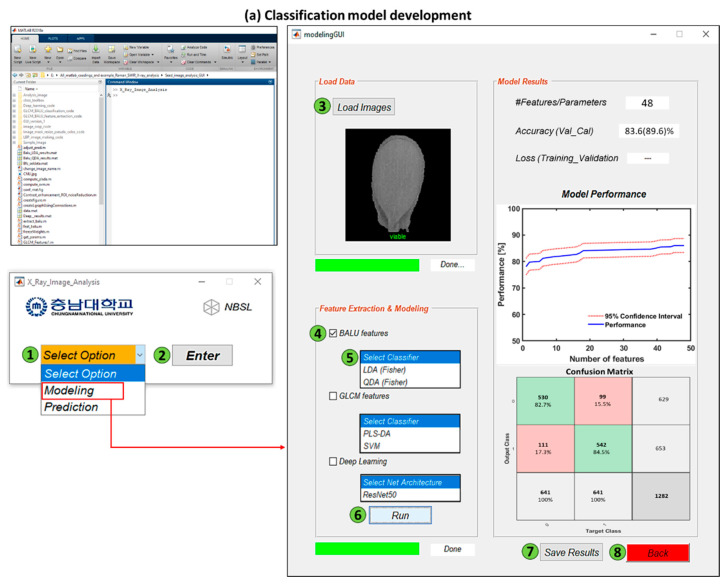

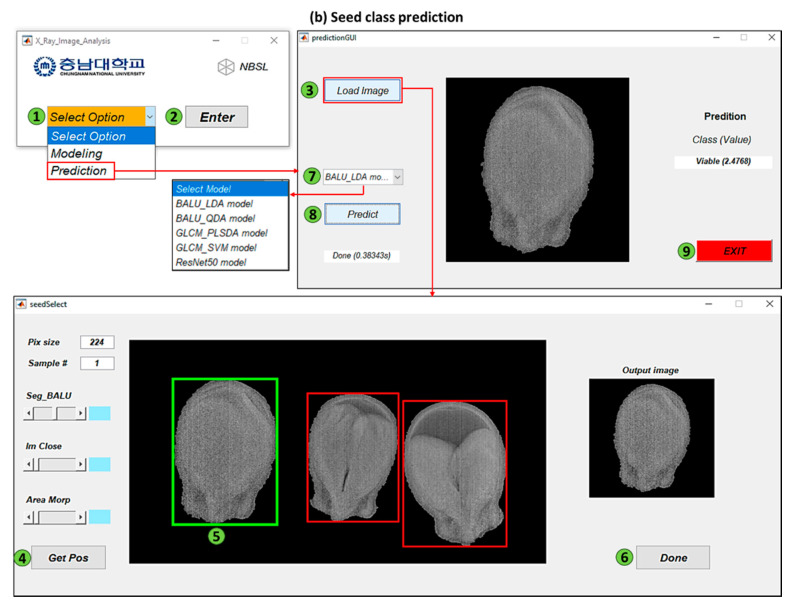
A custom-built software for classification (**a**) and prediction (**b**) of seed based on their morphology using X-ray imaging where green circles with number show the sequence of the operations.

A custom-built analysis software was developed for analyzing the X-ray images of seeds, classification model development, and applying the model to a new dataset for predicting the seed class (Figure 4). Different types of image features, such as LBP, Gabor, Haralic texture, local FFT, and gray level co-occurrence matrix features, could be extracted based on the type of seed and several classifiers, such as LDA, QDA, partial least squares-discriminant analysis (PLS-DA), support vector machine (SVM), and the ResNet50 architecture, could be tested. From the new input images of seeds, classes could be predicted by using the model data developed from the “Prediction” part.

## 3. Results and Discussion

### 3.1. Germination Test Result

**Figure 5:**
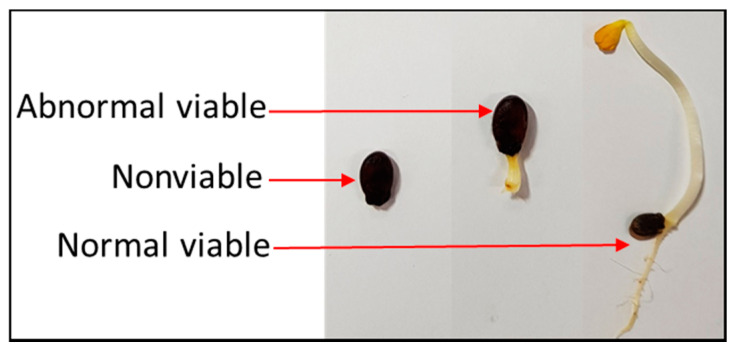
Types of seed found in germination test.

Three types of seeds were recognized by the germination test: (1) normal viable seeds—the straight radicle shape was observed, and the length was greater than 5 mm; (2) abnormal viable seeds—the radicle shape was twisted or had a terminal bud of less than 5 mm; and (3) nonviable seeds—no sign of germination (Figure 5). From the germination test outcome, the seeds were classified into two groups: normal viable seeds and nonviable/abnormal viable seeds.

In the germination test, a total of 1159 seed samples were classified as nonviable and abnormal viable and 641 seeds were normal viable seeds. The germination results are shown in Table 2. The results were used for supervised classification for both conventional machine learning and deep learning.

### 3.2. Conventional Machine Learning

**Figure 6:**
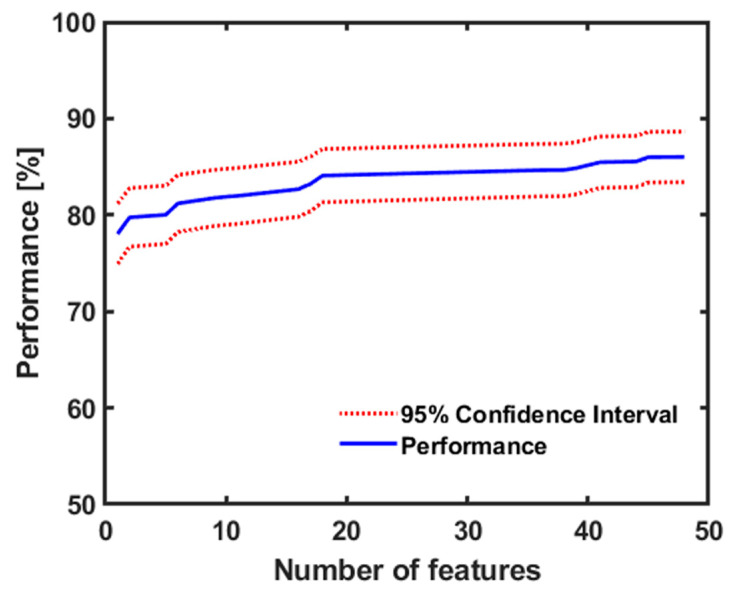
The performance of LDA classifier with the feature number.

For the conventional machine learning, the overall performances of tested classifiers are shown in Table 3. The best performance was obtained by the LDA classifier with an accuracy of 83.6% using 10-fold cross-validation. Generally, adding more feature numbers increases the classifier performance. However, excessive feature addition may extend analyzing time and biasness in the classification. Thus, selecting the optimum number of features was important, and in this case a total of 48 features was determined by the sequential forward selection (SFS) method and the Fisher score objective function for gaining the highest performance by LDA classifier. Figure 6 shows the correlation of the feature number and the classifier performance for the LDA classifier. Table 4 contains the feature names used in the LDA classifier to obtain the highest accuracy.

From the Table 4, it is observed that 87.5% features are from LBP, 4.2% from Gabor, and 6.2% from Fourier Ang (FFT) textural features contributed to obtain the best result. The normal viable seed contained a regular shaped endosperm where the intensity was uniform. In contrast, the nonviable and abnormal viable seeds’ endosperm accommodated air space or irregular size and shape. LBP features focused on the intensity difference both seed classes and distinguished the difference. Thus, LBP contributed the maximum feature number in the classification. A total of three sets of images were used for the classification where set_A, set_B, and set_C contributions are 39.6%, 20.8%, and 39.6%, respectively, for producing the LDA result. This result showed that set_A and set_C were important for the image enhancement operation.

### 3.3. Transfer Learning

In this study, five types of pre-trained networks were used: (1) simple ConvNet architectures, (2) AlexNet, (3) VGG-19, (4) ResNet-50, and (5) ResNet-101. The model was trained and validated using 10-fold random cross-validation with a total of 1282 (641 viable seeds and 641 nonviable and abnormal viable seeds integrated from three cultivars) images, where six types of augmentation were performed for each image (total of 7692). The validation and test group contained 15% each (total 30%) of the entire dataset. In terms of both the validation and test accuracy, ResNet-50 displayed a highest accuracy compare to the other ConvNets architectures. The performances of the pre-trained architectures are presented in Table 5.

**Figure 7:**
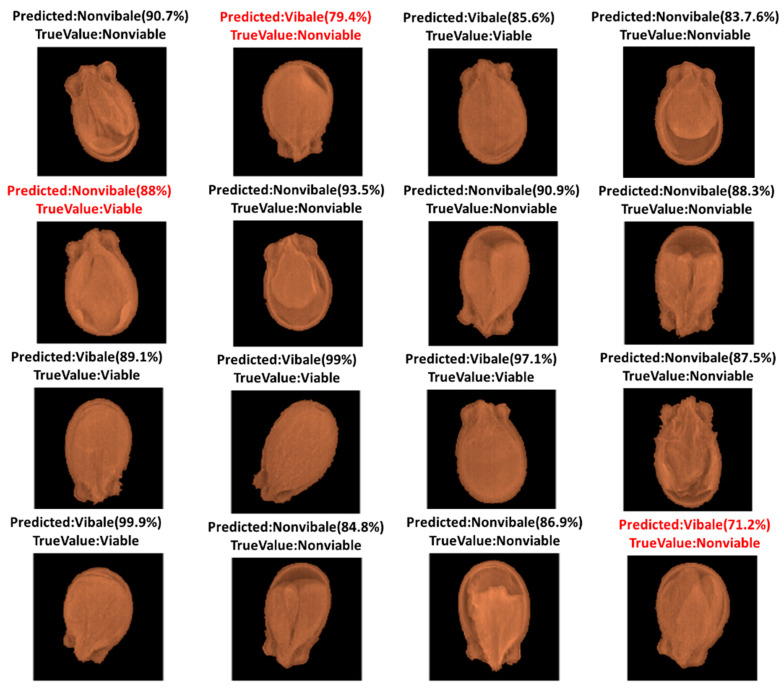
Randomly selected prediction class of the samples using ResNet50.

Figure 7 shows the prediction results from the test group for ResNet-50, where 16 samples were randomly selected to visualize the result. Three seeds were misclassified (texts are red colored) in the result. The misclassified seeds’ morphology was nominally deformed; thus, they were misclassified and this had on effect on the model’s accuracy. Dormant seeds, hard seeds, and chemically injured seeds failed to germinate, yet expressed marginal morphological deformation. X-ray imaging has the potential to analyze morphological conditions, rather than other types of nonphysical damage, causing the unsuccessful germination of seeds. Therefore, the developed model is more constructive when the seeds are externally or internally physically damaged, including broken or split seeds, an irregular shape of the endosperm, and empty or partially filled seeds.

In this study, the conventional classifier LDA showed the highest accuracy of 83.6%, which was lower than the pre-trained networks classification. In transfer learning, ResNet50 showed the best overall accuracy of 87.3%, which was the highest compared with other deep learning networks. ResNet-50 analyzed the image by applying 50 deep layers, which was more than the other networks except for ResNet-101, which was critical for learning the morphological image feature patterns in the viable and nonviable seeds and classifying them with a high accuracy. ResNet-101 contains 101 deep lays which may be arbitrary for two class classification and produce similar results following other architectures. However, the function of each layer of the deep learning architecture is not yet fully understood, and deep networks are still seen as a “black-box” [20]. Understanding the details of the functions of each layer is, therefore, a topic for future study. Simple ConvNets, AlexNet, and VGG-19 required a shorter training time than ResNet-50 and ResNet-101 as they used fewer layers. Hasan et al. (2016) reported that the performance of ConvNets is affected by the number of classes [32]. A lower number of classes can improve the performance of ConvNets. In this study, only two classes (viable and nonviable) were considered to be identified, which were fitted for the used ConvNets and showed a good performance compared to conventional machine learning.

Several factors, such as the temperature, moisture, storage time, seed borne mycoflora, and fungi, affect the deterioration of seed components during harvest and post-harvest periods [21]. In spite of careful handling, seed cover can be easily damaged during shipment and germs can enter the seed. A high temperature reduces the moisture content of the cotyledons, resulting in deformation of their normal shape and increasing the air cavity inside the seed. Lipid autoxidation is an inevitable process that occurs during the storage period of a seed and increases the brittleness of the cell structure as a result of membrane fluidity reduction and elasticity loss in internal proteins [33]. This strand also plays an important role in the morphological alternation of seeds.

**Figure 8:**
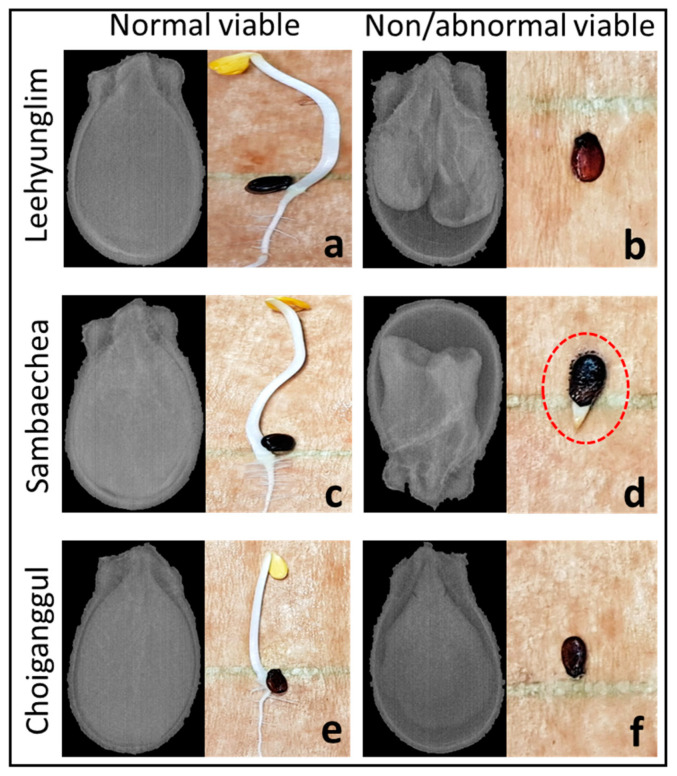
Morphological difference in normal viable and nonviable and abnormal viable seed in 3 watermelon seed cultivars. Ehiongrim and Samveshay cultivars showed distinct morphological difference than Suyegangkul. Seeds contained good shaped internal structures and produced healthy seedlings from all cultivars shown in (**a**,**c**,**e**). Poor internal structure seeds failed to germinate (**b**,**f**) or produce abnormal seedlings (**d**) (no proper root and shoot, mark with red dotted circle).

The internal deterioration of seeds highly depends on the seed cultivar (Figure 8). In this study, out of the three cultivars used, two cultivars (Leehyunglim and Sambaechea) showed high variation in the morphological pattern of the normal viable and nonviable and abnormal viable seeds (Figure 8a–d). In contrast, for the Choiganggul cultivar, a nominal distinction was found in the internal structure of seeds for normal viable and nonviable/abnormal viable seeds (Figure 8e,f). With a minimum effect on the morphological structure, chemical alternations sometimes can occur in the seed components due to inappropriate environmental conditions and long-term storage, which may cause unsuccessful germination. This scenario was found to be true for the Choiganggul cultivar and caused misclassification in the model performance.

X-ray imaging has the potential to evaluate the morphological damage, but is limited to inspecting chemical degradation, genetic effects, dormancy, and hard seeds. Therefore, the model is suitable when morphological alternations are perceptible in the seed structure. The developed model can also be employed to investigate external damages, such as a broken or split seed coat, which causes rapid internal degradation. Internal defects of seeds are invisible and hard to detect without the aid of an instrument; hence, an X-ray imaging-based classification model can be a feasible solution for overcoming the difficulty in inspecting the internal defects of seeds. Although the classification algorithm applied for this study was developed using an off-line X-ray imaging system, the combined system could nevertheless be used to build an online non-invasive quality sorter using an online-based X-ray system. The training and application of deep neural networks requires high-performance hardware systems (CPU, GPU, RAM) on a computer, which is a limiting factor [34]. To address this limitation, several cloud services, such as Amazon Elastic Compute Cloud and Paperspace GPU Cloud, can be used as an alternative for acquiring high-performance computing hardware on-site [20].

X-ray projection imaging is remarkably fast in terms of acquiring images compared to CT imaging. In addition, the exposure time in X-ray imaging is also shorter than that in CT imaging, which is favorable, as the shorter time of exposure means that the seed samples receive less X-ray emission. A long exposure time may cause physical or nonphysical alternations in the seed properties, which may cause unsuccessful germination. Although the classification and prediction algorithm were developed using an offline X-ray system, the combined system could be used to construct an online non-invasive quality sorter by adopting an online X-ray machine.

## 4. Conclusions

X-ray imaging is a potential technique for inspecting the quality of seeds based on their internal condition. Rapid image acquisition and the shorter exposure time of X-ray projection imaging are advantageous for the speedy inspection of seed samples. In this study, classification models were developed with conventional machine leaning, as well as deep learning, to compare their performances. The acquired X-ray images were enhanced with image processing algorithms, such as intensity and contrast enhancement, and noise reduction before image feature extraction. Among the conventional classifiers, the LDA method showed the highest accuracy (83.6%) with 10-fold cross-validation. The performance of transfer learning, simple ConvNets, AlexNet, VGG-19, ResNet-50, and ResNet-101 architectures modified to train the morphological patterns of seeds demonstrated a higher accuracy than that of conventional methods. ResNet-50 performed (87.3%) better than other ConvNets architectures. The models of this study were developed using three watermelon seed cultivars, but the methodology can be applied broadly for other kinds of seeds such as muskmelon and cucumber seed. Even though X-ray images are suitable for evaluation of morphological alternations inside the seed, chemical alternations in the seed’s property, genetic effects, and dormancy, which also provoke unsuccessful germination, are limited to evaluation by X-ray imaging. However, the morphological diversity is eventually visualized if the chemical or other alterations in the seed are pronounced. The X-ray image analysis model developed in this study can be used as a feasible tool to inspect the seed quality based on the seed morphology, which is beneficial for both seed growers and companies.

## Figures and Tables

**Table 1:** Image acquisition parameters for X-ray projection imaging.

Parameters	Values
System	Xeye-5100F
Source voltage	50 kV
Source current	100 μA
Exposure time	0.05 s
Magnification	18×
Filter	Glass effect

**Table 2:** Germination test result of the seed samples.

Cultivars	Viable Seed	Nonviable Seed	Total	Germination Rate
Leehyunglim	72	528	600	12%
Sambaechea	116	484	600	19%
Choiganggul	453	147	600	76%
Overall	641	1159	1800	36%

**Table 3:** Conventional machine learning performance.

Region of Interest (ROI)	Classifier	2-Classes Performance (%)		
		Mean	UCI ^a^	LCI ^b^
Whole seed	LDA	83.6	86.1	81.1
	QDA	80.8	84.4	77.2
	KNN (K = 5)	63.7	72.6	54.9

^a^ Upper confidence interval (UCI); ^b^ Lower confidence interval (LCI). LDA: linear discriminant analysis; QDA: quadratic discriminant analysis; KNN: k-nearest neighbors algorithm.

**Table 4:** The selected features used by LDA classifier applying the sequential forward selection (SFS) method and the Fisher score objective function.

Number	Features Name	Number	Features Names
1	i-Gabor(1,1)[Max-A]	25	i-LBP(2,2)[8,u2][sd-C]
2	i-LBP(3,34)[8,u2][sd-C]	26	i-LBP(2,26)[8,u2][sd-A]
3	i-LBP(2,53)[8,u2][sd-B]	27	i-LBP(2,29)[8,u2][Max-B]
4	Fourier Ang (2,1)[rad][Max-C]	28	i-LBP(1,36)[8,u2][sd-C]
5	i-LBP(3,44)[8,u2][sd-A]	29	i-LBP(4,42)[8,u2][sd-A]
6	Fourier Abs (1,1)[Max-C]	30	i-LBP(3,51)[8,u2][sd-C]
7	i-Gabor-J[sd-A]	31	i-LBP(2,6)[8,u2][sd-A]
8	i-LBP(3,27)[8,u2][sd-A]	32	i-Intensity Skewness[Max-C]
9	i-LBP(3,58)[8,u2][Max-A]	33	i-LBP(3,2)[8,u2][sd-C]
10	i-LBP(1,10)[8,u2][Max-C]	34	i-LBP(3,41)[8,u2][Max-C]
11	i-LBP(4,57)[8,u2][sd-C]	35	i-LBP(1,57)[8,u2][Max-A]
12	i-LBP(3,56)[8,u2][Max-C]	36	i-LBP(1,5)[8,u2][Max-A]
13	i-LBP(1,52)[8,u2][sd-C]	37	i-LBP(1,30)[8,u2][sd-B]
14	i-LBP(1,38)[8,u2][sd-A]	38	i-LBP(1,51)[8,u2][Max-A]
15	i-LBP(1,46)[8,u2][sd-C]	39	i-LBP(2,57)[8,u2][Max-B]
16	i-LBP(3,12)[8,u2][sd-C]	40	Fourier Ang (2,2)[rad][Max-C]
17	i-LBP(3,2)[8,u2][Max-A]	41	i-LBP(3,15)[8,u2][Max-A]
18	i-LBP(2,30)[8,u2][sd-A]	42	i-LBP(1,37)[8,u2][sd-A]
19	i-LBP(2,46)[8,u2][sd-A]	43	i-LBP(1,50)[8,u2][sd-B]
20	i-LBP(4,13)[8,u2][sd-B]	44	i-LBP(4,21)[8,u2][sd-C]
21	i-LBP(2,20)[8,u2][sd-B]	45	i-LBP(2,35)[8,u2][sd-A]
22	i-LBP(2,20)[8,u2][sd-A]	46	i-LBP(1,8)[8,u2][Max-C]
23	i-LBP(2,48)[8,u2][sd-C]	47	i-LBP(3,34)[8,u2][sd-B]
24	i-LBP(1,29)[8,u2][sd-B]	48	i-LBP(3,7)[8,u2][sd-B]

i-LBP(d,h): local binary patterns. Where d is the number of compared pixels with h—neighboring pixels. i-Gabor(a,b): Gabor filters, where a is the frequency number, and b is the number of orientations. Fourier Ang (FFT) (u_0_,T_0_)-(Abs, Rad): Fourier-Based textural features, where u_0_ is the frequency number, and T_0_ is the period. The information in the last brackets [] are the different quality enhanced images used for extracting features.

**Table 5:** Classification performance of simple ConvNet, AlexNet, VGG-19, ResNet-50, and ResNet-101 architectures for seed quality inspection based on morphology. The validation accuracy was obtained by using 10-fold cross-validation.

Network Architecture	Classification Accuracy	
	Validation Accuracy (%)	Test Accuracy (%)
Simple ConvNets	88.7	84.5
AlexNet	92.1	86.4
VGG-19	91.2	86.9
ResNet-50	92.5	87.3
ResNet-101	91.9	86.6
